# Somatic Variants Acquired Later in Life Associated with Thoracic Aortic Aneurysms: *JAK2* V617F

**DOI:** 10.3390/genes15070883

**Published:** 2024-07-05

**Authors:** Christina Waldron, Mohammad A. Zafar, Deqiong Ma, Hui Zhang, Daniel Dykas, Bulat A. Ziganshin, Andreea Popa, Alokkumar Jha, Jennifer M. Kwan, John A. Elefteriades

**Affiliations:** 1 Aortic Institute at Yale-New Haven Hospital, Yale University School of Medicine, New Haven, CT 06510, USA; christina.waldron@yale.edu (C.W.); mohammad.zafar@yale.edu (M.A.Z.); bulat.ziganshin@yale.edu (B.A.Z.); 2 DNA Diagnostics Lab, Yale University School of Medicine, New Haven, CT 06510, USA; deqiong.ma@yale.edu (D.M.); hui.zhang@yale.edu (H.Z.); daniel.dykas@yale.edu (D.D.); 3 Department of Pathology, Microbiology and Immunology, Vanderbilt University Medical Center, Nashville, TN 37232, USA; andreea.popa@vumc.org; 4 Centre for Neurogenetics, Weill Cornell Medicine, New York, NY 10021, USA; alokkumar.jha@yale.edu; 5 Section of Cardiovascular Medicine, Yale University School of Medicine, New Haven, CT 06510, USA; jennifer.kwan@yale.edu

**Keywords:** thoracic aortic aneurysm, *JAK2* V617F variant, cardiovascular genetics

## Abstract

The *JAK2* V617F somatic variant is a well-known driver of myeloproliferative neoplasms (MPN) associated with an increased risk for athero-thrombotic cardiovascular disease. Recent studies have demonstrated its role in the development of thoracic aortic aneurysm (TAA). However, limited clinical information and level of *JAK2* V617F burden have been provided for a comprehensive evaluation of potential confounders. A retrospective genotype-first study was conducted to identify carriers of the *JAK2* V617F variant from an internal exome sequencing database in Yale DNA Diagnostics Lab. Additionally, the overall incidence of somatic variants in the *JAK2* gene across various tissue types in the healthy population was carried out based on reanalysis of SomaMutDB and data from the UK Biobank (UKBB) cohort to compare our dataset to the population prevalence of the variant. In our database of 12,439 exomes, 594 (4.8%) were found to have a thoracic aortic aneurysm (TAA), and 12 (0.049%) were found to have a *JAK2* V617F variant. Among the 12 *JAK2* V617F variant carriers, five had a TAA (42%), among whom four had an ascending TAA and one had a descending TAA, with a variant allele fraction ranging from 11.2% to 20%. Among these five patients, 60% were female, and average age at diagnosis was 70 (49–79). The mean ascending aneurysm size was 5.05 cm (range 4.6–5.5 cm), and four patients had undergone surgical aortic replacement or repair. UKBB data revealed a positive correlation between the *JAK2* V617F somatic variant and aortic valve disease (effect size 0.0086, *p* = 0.85) and TAA (effect size = 0.004, *p* = 0.92), although not statistically significant. An unexpectedly high prevalence of TAA in our dataset (5/594, 0.84%) is greater than the prevalence reported before for the general population, supporting its association with TAA. *JAK2* V617F may contribute a meaningful proportion of otherwise unexplained aneurysm patients. Additionally, it may imply a potential JAK2-specific disease mechanism in the developmental of TAA, which suggests a possible target of therapy that warrants further investigation.

## 1. Introduction

Thoracic aortic aneurysms (TAAs) often grow asymptomatically before rupturing and have an incidence of 5.3 per 100,000 individuals/year [1]. Due to the devastating morbidity and mortality associated with TAAs and dissections, medical science has long sought to identify genetic etiologies of TAA in order to improve identification of asymptomatic patients and management of aortic disease on a gene-by-gene basis [2,3,4,5].

Historically, syndromic connective tissue diseases were among the first recognized drivers of TAAs [6]. Later, non-syndromic familial etiologies explained an additional 20% of TAAs, with a predominantly autosomal dominant and genetically heterogenous inheritance [7,8,9,10]. Despite these advances, two-thirds of TAAs occur without known genetic etiology or explanation. Non-genetic risk factors for TAAs include cystic medial degeneration, which weakens the aortic wall and promotes dilatation and aneurysm formation [11]. Atherosclerosis is infrequently the driver of ascending TAAs, being predominantly associated with abdominal aortic aneurysms [11,12].

Population-scale genomic sequencing continues to deepen our understanding of both germ line (inherited) and somatic (acquired in cells later in life) variants and their roles in cardiovascular disease [10]. In particular, the *JAK2* V617F somatic variant is frequently identified as a driver in adult-onset myeloproliferative neoplasms and is increasingly being associated with cardiovascular complications, including thoracic aortic aneurysms [13,14,15]. Additionally, subjects with clonal hematopoiesis of indeterminate potential (CHIP) with the *JAK2* V617F somatic variant had a >12-fold increase in the risk of TAA (but not abdominal aortic aneurysms) compared to those without *JAK2* V617F CHIP [15,16,17,18]. Herein, we report an independent dataset of *JAK2* V617F somatic variants which further validates their association with the development of TAA.

## 2. Materials and Methods

We conducted a genotype-first retrospective study to identify *JAK2* V617F carriers from the internal database of exome sequencing in the Yale DNA Diagnostics Lab. DNA was extracted from peripheral blood and saliva. This in-house exome database containing 12,349 clinical-grade exomes from a broad phenotypic spectrum was interrogated. A total of 594 patients (594/12349:4.8%) were referred for genetic evaluation of TAA, not including patients who were referred specifically for FBN1 testing. Electronic medical records were queried to investigate patient demographics, medical and surgical histories, and relevant imaging studies.

To compare the allele frequency of the V617F variant in our dataset to the general population, the gnomAD dataset v4 was utilized. Similar to our database, DNA was derived from peripheral blood with a small portion extracted from other sources, such as saliva or buccal swabs. The best practice GATK pipeline was adopted for variant calling. Additionally, to understand the prevalence of the *JAK2* V617F somatic variant, we investigated the overall incidence of somatic variants in the *JAK2* gene across various tissue types in the healthy population based on reanalysis of SomaMutDB [19]. To further analyze the *JAK2* V617F somatic variant prevalence, we utilized summary statistics from the Neale Lab data from the UK Biobank (UKBB) [20,21] cohort, including 416,555 individuals, and FinnGen data, including 412,181 samples. Using these data, individuals with the *JAK2* V617F variant who presented with a circulatory system disease were compared to a control group of individuals without that disease. Logistic regression models were used to estimate the odds ratios for the association between *JAK2* V617F and the disease, adjusting for potential confounders, such as age, sex, and other relevant covariates. The effect sizes were calculated from the regression coefficients, representing the likelihood of disease presence given the *JAK2* V617F variant.

## 3. Results

### 3.1. Patient Characteristics

A total number of 12 carriers of the *JAK2* V617F somatic variant were identified from our database. Among them, five manifested TAA (42%), with variant allele fractions (VAFs) ranging from 11.2–20%. The remaining 7 patients with a *JAK2* V617F somatic variant were not found to have a TAA, and 589 patients with a TAA did not have a *JAK2* V617F somatic variant. Patient characteristics and relevant medical histories are presented in Table 1. Four patients had an ascending TAA, one had a descending TAA, 60% were female, and the average age at diagnosis was 70. Mean height and weight were 1.76 m and 92.9 kg, respectively. Mean ascending aneurysm size was 5.05 cm (range 4.6–5.5 cm), and four patients had undergone surgical aortic replacement or repair. Each patient had some history of cardiovascular disease; four patients had hypertension, three had atrial fibrillation, one had a prior coronary artery bypass surgery, and two had coronary artery disease (Table 1).

### 3.2. Patient 1

This 76-year-old female with a history of hypertension and gastroesophageal reflux disease presented to our tertiary care center with weakness and lightheadedness, which were attributed to atrial fibrillation of unclear chronicity. She also manifested sinus bradycardia. Upon admission, she was noted to have exertional chest pain, responsive to nitroglycerin. Importantly, her transthoracic echocardiogram demonstrated an ATAA (4.9 cm), later confirmed on Computed Tomographic Angiography at 4.9–5.1 cm in diameter.

She safely underwent planned surgical intervention with ascending/hemi arch replacement, sinus of Valsalva reconstruction, and 3-vessel coronary artery bypass graft. There were no intraoperative complications, and the patient did well post-operatively. Genetic testing revealed a pathogenic *JAK2* V617F somatic variant. VAF was 11.5%.

### 3.3. Patient 2

This 71-year-old male with a history of hypertension, atrial fibrillation, coronary artery disease with chest discomfort (without ischemia on a stress test), hyperlipidemia, and prior porcine aortic valve replacement in 2006 was found to have progressive ascending aortic dilatation, reaching a maximal diameter of 5.2 cm, along with moderate stenosis of the bioprosthetic aortic valve.

The patient underwent redo aortic valve replacement and replacement of the ascending aorta. There were no intraoperative complications, and the patient did well post-operatively. Genetic testing incidentally noted a *JAK2* V617F somatic variant in peripheral blood analysis. VAF was 16%.

### 3.4. Patient 3

This 79-year-old female with a history of hypertension underwent an echocardiogram after a fall, which demonstrated a dilated ascending aorta. CT scan demonstrated a moderate-sized ATAA (4.8 cm) and a healed prior descending aortic dissection. Due to concern for potential ascending aortic dissection, the patient underwent elective surgical repair. There were no intraoperative complications, and the patient did well post-operatively. Genetic testing incidentally noted a *JAK2* V617F somatic variant in peripheral blood analysis. VAF was 17.2%.

### 3.5. Patient 4

This complex 49-year-old female presented with a history of acute myeloid leukemia, polycythemia vera, hypopituitarism, postural hypotension, and spontaneous colon rupture. She had a personal and family history of thoracic aortic aneurysms but no known ATAA variant. She presented to our tertiary care center with left foot bruising and intermittent abdominal pain and nausea. On exam, she manifested pectus excavatum, scoliosis, and easy bruising. While in the emergency room, she experienced back pain and orthostatic hypotension. Given the history of aortic aneurysms, for which she had been followed by cardiac surgery at an outside institution, she underwent a CT scan, which demonstrated increased aneurysmal dilation of the ascending aorta, from 4.8 cm to 5.1 cm. She was admitted for observation due to the enlarging aorta and polycythemia vera. We embarked on a course of non-operative serial imaging because of worrisome comorbidities. A *JAK2* V617F variant with a VAF of 20% was identified around the time of her diagnosis with polycythemia vera. Her ATAA has continued to be closely monitored, without intercurrent adverse aortic event. 

### 3.6. Patient 5

This 75-year-old male with a history of hypertension, hyperlipidemia, atrial fibrillation, venous thrombosis, and coronary artery disease presented to our tertiary care center with acute chest pain and worsening back pain. CT scan demonstrated a type B dissection from the proximal descending aorta to the diaphragm. The patient was managed medically, followed by vascular surgery. A follow-up CT scan demonstrated significant dilation and ulceration along the descending thoracic aorta, and the patient underwent a planned endovascular repair of the descending thoracic aorta. There were no intraoperative complications, and the patient did well post-operatively. Genetic testing noted a *JAK2* V617F somatic variant in peripheral blood analysis with a VAF of 18%.

### 3.7. Correlation of Variant Allele Fraction and Thoracic Aortic Aneurysm

A comparison of VAFs between carriers of TAA (N = 5) and carriers without TAA (N = 7) was performed. The mean variant allele fraction for patients with the *JAK2* V617F and TAAs was lower at 16.58% (SD: 2.86%, 95% CI: 12.61%, 20.55%) compared to 26% (SD: 10.43%, 95% CI: 15.58%, 36.42%) for patients with *JAK2* V617F variant and no TAA (*p* = 0.1), although this difference is not clinically significant given the small sample size.

### 3.8. TAA Prevalence and Somatic Variant Distribution

Among the 12 *JAK2* V617F somatic variant carriers in our internal database, four presented with an ATAA. One additional carrier presented with a type B aortic dissection. The additional *JAK2* V617F somatic variant carriers in our database who did not have a TAA were between 58- to 83-years-old, and clinical features included muscle pain and weakness, venous thrombosis, myelofibrosis, polycythemia vera, stroke, cardiomyopathy, and prostate cancer (Table 2). The prevalence of the *JAK2* V617F somatic variant among the ATAA (ascending) patients was 4/594 (0.67%) and 5/594 (0.84%) among all patients with TAA (ascending and descending). In contrast, the prevalence of this somatic variant in our exome database was approximately 6.9–8.6-fold lower, at 12/(12,349 × 2) (0.049%).

In the general population (gnomAD v4 including 730,947 exomes and 76,215 whole genomes), the lumped allele frequency of this variant, including somatic and possible germline changes, is approximately 471/1,605,918 (0.029%) with allelic fractions from 20% to >85% (age range: <30-year-old to >80-year-old), which is about 14 times lower than the allele frequency in our TAA group (5/(594 × 2): 0.42%) [22]. The prevalence of the V617F variant ranges from 0.023% in individuals 50–55 years old, to 0.066% in individuals 60–65 years old, to a maximum allele frequency of 0.14% in individuals 70–75 years old [22]. These prevalences are still approximately 3 to 18 times lower than those seen in our ATAA/TAA group. Somatic variants in *JAK2* V617F from a study using the UKB were also interrogated with a prevalence of 0.04% of the entire 454k cohort and made up 0.66% of those who had clonal hematopoiesis of indeterminate potential.

**Figure 1:**
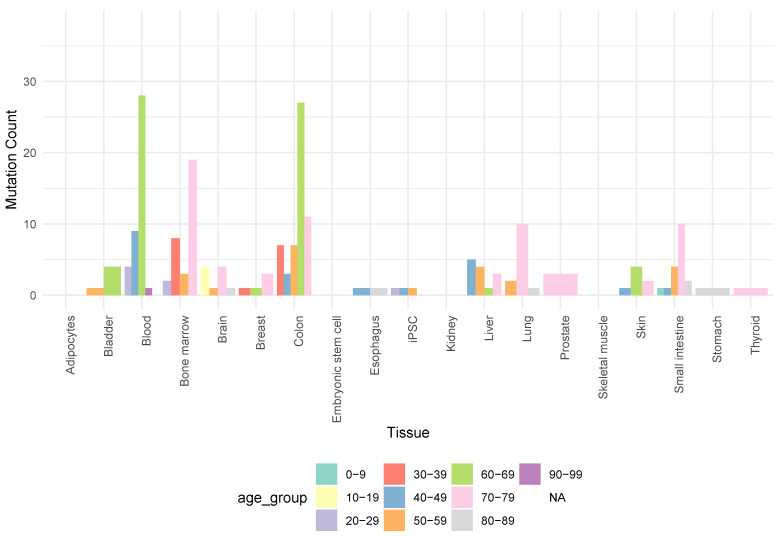
Somatic Variant Burden by Tissue and Age Group for JAK2. This figure shows the incidence of the somatic *JAK2* variant in various tissue types across the healthy population based on a reanalysis of SomaMutDB.

Our findings reveal that somatic mutations in *JAK2* begin to appear between the ages of 20–29, with a significant increase in individuals over the age of 40, and the highest number of somatic changes observed in the 60–69 age group based in a healthy control cohort (Figure 1). Further analysis of *JAK2* V617F somatic variant prevalence utilizing UKBB and FinnGen data is highlighted in Table 3, which presents the effect of *JAK2* V617F on circulatory system phenotypes with the number of samples being included in each category and shows a positive effect size with aortic valve disease and thoracic aortic aneurysms (effect size = 0.0086 and effect size = 0.004, respectively), although this was not statistically significant. *JAK2* V617F somatic variants exhibited a negative effect size for abdominal aortic aneurysms (effect size = −0.026) and atherosclerosis of the aorta (effect size = −0.034, although not statistically significant).

## 4. Discussion

Here, we report five patients with *JAK2* V617F somatic variants and TAAs. A significantly high occurrence (42%) of TAAs were found in this case series. This percentage is consistent with but higher than the result from a previous study among Japanese patients with V617F-related MPN (23%) [13]. We also observed an increased prevalence of thoracic aortic aneurysms in the UKBB and FinnGen data among carriers of the *JAK2* V617F variant (effect size 0.004). These findings—our five *JAK2* V617F somatic variant carriers with TAA and the UKBB data showing increased TAA prevalence with *JAK2* V617F—suggest a possible JAK2-specific mechanism attributable to TAA after adjustment of known clinical risk factors for TAA.

The somatic *JAK2* V617F variant has a central role in the pathogenesis of Philadelphia negative (Ph-) myeloproliferative neoplasms (MPN), including essential thrombocythemia, polycythemia vera, and primary myelofibrosis [23]. It is also found in patients with different types of venous thromboses without an overt chronic MPN [24]. Even though it can be carried by healthy individuals [25,26], one study found that 76.2% (48 out of 63) of carriers were eventually diagnosed with MPN [27]. It has also been associated with an increased risk for vascular complications, including coronary heart disease [28,29,30]. More recently, a cross-sectional study among 39 MPN patients with *JAK2* V617F found that nine patients (23%) exhibited the presence of thoracic (7/9) and/or abdominal aortic aneurysms (2/9). Follow-up functional study on transgenic (JAK2^V617F^) mice further demonstrated a crucial role of hematopoietic *JAK2* V617F in the development of aortic aneurysms [13].

A total of 30% of TAA cases can be “explained” on the basis of inherited mutations or suspicious inherited genetic variants, with somatic variants potentially “explaining” another subset, as in the patients reported here [6,7,8,9,10]. Furthermore, the cadre of patients with somatic *JAK2* V617F variants and associated TAA may not be trivial, especially in older patients known to be subject to acquired hematologic variants later in life. With genetic sequencing becoming commonplace, other mosaicisms may be uncovered, and somatic variants may need to be investigated more regularly in TAA patients. Importantly, this novel genetic mechanism suggests the potential for utilizing hematopoietic JAK–STAT signaling as a target of therapy for TAA.

A recent paper suggests that a JAK2-related pro-inflammatory state may be damaging to the aorta and ultimately lead to acute type A aortic dissection [31], as in our Patient #3. The *JAK2* V617F variant has previously been shown to be associated with vascular complications, including coronary artery disease, peripheral artery disease, and abdominal aortic aneurysms [13,14,32,33]. One potential pathogenetic mechanism underlying this association postulates that *JAK2* V617F-positive leukocytes upregulate matrix metalloproteinase-9, which is known to be associated with the development of atherosclerosis [13]. Additional studies have underscored a causal relationship between the *JAK2* V617F mutation and vascular disease through upregulation of pro-inflammatory macrophages via dose-response relationships in mouse models [16]. Promotion of aortic dissection in a murine model has also been reported [34]. However, many of these studies do not differentiate between segments of the aorta, which are understood to behave as distinct disease entities, with the ascending aorta demonstrating degenerative pathology, whereas the descending and abdominal aorta demonstrates inflammatory pathology [12].

Blood somatic variants, including those in *JAK2* V617F, have a known propensity to increase with age. However, data from SomaMutDB show that the *JAK2* somatic variant burden in blood spans a range of ages from 20 to 99 years. These data suggest that although the prevalence of the *JAK2* V617F somatic variant increases with age, a considerable variant burden has been shown in younger ages between the years of 20 and 49. Furthermore, we investigated two independent cohorts from the UKBB and FinnGen to identify the association of *JAK2* V617F with aortic diseases. Both UKBB and FinnGen show a strong correlation of *JAK2* V617F with myeloproliferative disease and polycythemia vera. Our analysis identified a small positive effect of the *JAK2* V617F variant on nonrheumatic aortic valve disorders (effect size = 0.05) and aortic valve disease (Effect Size = 0.0086). Previous studies have indicated a strong link between aortic valve disease and the prevalence of TAA [35,36]. These findings suggest a potential contributory role of the mutation in the pathogenesis or progression of aortic valve-related abnormalities. Although the effect sizes are small, they provide a preliminary basis to hypothesize that *JAK2* V617F may influence the cellular pathways involved in valve function or integrity. Conversely, the variant exhibited negative effect sizes for abdominal aortic aneurysm (Effect Size = 0.026) and atherosclerosis of the aorta (Effect Size = 0.034), although not statistically significant. These negative associations warrant further investigation to better understand its potential protective effect against these abdominal aortic conditions.

Additionally, our analysis investigated the broader category of thoracic aortic aneurysms, where a very small positive effect size was noted (Effect Size = 0.0040). However, a somatic variant condition, such as *JAK2* V617F CHIP, was shown to increase the risk of TAA by more than 12-fold [15]. Taken together, this opens up discussion for potential influences of the *JAK2* V617F variant on the aneurysmal disease processes in the thoracic aorta, possibly through modulation of vascular smooth muscle cell function or response to mechanical stress. Thus, our comparison underscores the significant role of the *JAK2* V617F variant as a potent risk factor for aortic disease, ultimately leading to TAA.

While the effect sizes reported are small, even modest increases in effect size can be significant in the context of complex diseases, like thoracic aortic aneurysm. Small effect sizes can indicate subtle but meaningful contributions to disease risk, especially when considering multifactorial etiologies where multiple small effect sizes collectively contribute to a clinically significant outcomes [37,38,39,40,41]. Therefore, the presence of a small positive effect size for thoracic aortic aneurysms, alongside our findings in the internal database, supports further investigation into the role of the *JAK2* V617F somatic variant in aortic disease.

Direct or indirect associations are crucial in understanding disease mechanisms. Direct associations can reveal specific causal links, while indirect associations can highlight broader pathogenic processes involving multiple intermediate steps. In the case of *JAK2* V617F, the somatic variant may not directly cause thoracic aortic aneurysms but could contribute to conditions that predispose individuals to this disease. The pro-inflammatory state linked to *JAK2* V617F could be a contributing factor in the development of TAAs, emphasizing the importance of considering both direct and indirect pathways in disease association studies [34,42].

Somatic variants are heterogeneous and may be due to a variety of external factors, including environmental influences or therapeutic interventions. These factors have the potential to alter the mutational landscape and contribute to a higher burden of variants, which may be the case in the 49-year-old patient described here (Patient #4). This association would suggest that the presence and burden of the *JAK2* V617F variant may be a direct response to such external stresses and requires further investigation. The prevalence of the *JAK2* V617F variant in our exome database was 0.049%, which is lower than the range previously reported [27,43]. However, this may be due to population differences in risk factors, such as age and smoking [44].

The significantly high prevalence of TAAs among our database of *JAK2* V617F carriers raises the important question of whether echocardiography should be considered as regular surveillance among patients found to have the *JAK2* V617F variant. Additionally, every patient had a history of cardiovascular disease; four patients had hypertension, three had atrial fibrillation, one had a prior coronary artery bypass surgery, and two had coronary artery disease. Given the small sample size of our study, no obvious correlation was observed between allelic fraction and thoracic aortic aneurysm size; however, this potential association should be assessed in a study with a larger sample size. These findings further suggest the importance of cardiovascular surveillance among patients with the *JAK2* V617F variant.

The allele frequency of the *JAK2* V617F variant in our database is relatively higher than that in gnomAD v2 and v4. This difference could be partially due to sample size-, age-, and ethnicity-related sampling biases as well as the parameter setting for somatic variant calling. Meanwhile, our dataset is also a convenient dataset of patients with a variety of phenotypes, including some patients with hematological diseases. We did not observe a significant difference in variant allele fractions between patients who were *JAK2* carriers with TAA and those without TAA, though our dataset is small.

Somatic variants remain under-investigated in terms of potential contributions to the reservoir of TAA patients. Thus far, even when considering both causative mutations and suspicious variants, only about 30% of TAA can be “explained” on a genetic basis [6,7,8,9,10]. It is possible that somatic variants (in hematopoietic cells or even in aortic smooth muscle cells themselves) may narrow the gap of genetically “unexplained” cases of TAA.

### Limitations

The number of patients in this real-world study is small, as recognition of the role of somatic variants in TAA is only now emerging. A strength of the present study is the detailed clinical profile, report of therapy, and follow-up with a granularity not always achievable in emerging large database reports [15]. The effect sizes were small; however, thoracic aortic aneurysms are a complex disease potentially involving multifactorial etiologies. The *p*-values associated with the effect sizes of *JAK2* V617F on circulatory system diseases were large. However, the control cohort sizes from the UKBB and FinnGen databases were sufficiently large to substantiate the significance of the observed effect sizes. The gnomAD dataset is a useful, publicly available resource, but there is an under-representation of non-European populations. Similarly, the majority of participants within the UK Biobank and FinnGen are of Caucasian background, which may limit the generalizability of these results.

## 5. Conclusions

An unexpectedly high prevalence of V617F carriers among our dataset of patients with TAA further supports its association with TAA. This *JAK2* V617F variant may contribute to a meaningful proportion of otherwise unexplained patients with TAAs. This finding may also imply a potential JAK2-specific disease mechanism in the development of TAA besides the possibly increased risk for athero-thrombotic cardiovascular disease caused by *JAK2* V617F. The exact mechanism warrants further investigation. These findings call for a larger-scale comparison carried out among age-matched study populations after proper adjustment of gender and other potential CHIP-related cardiovascular risk factors.

## Figures and Tables

**Table 1:** Characteristics of Patients with JAK2-V617F Somatic Variant and Thoracic Aortic Aneurysms.

Characteristic	Patient 1	Patient 2	Patient 3	Patient 4	Patient 5
Sex	Female	Male	Female	Female	Male
Age	76	71	79	49	75
Weight (kg)	80.7	100.5	69.3	112	102.1
Height (m)	1.6	1.83	1.7	1.88	1.78
Aneurysm Size (cm)	4.9 (asc)	5.2 (asc)	4.6 (asc)	5.5 (asc)	4.2 (desc)
JAK2 V617F Level in Blood	11.5%	16%	17.2%	20%	18%
Family History	Unknown	Unknown	Unknown	Sister—4 cm Asc AA	Unknown
Smoking History	Never	Former	Former	Never	Former
Diagnostic Imaging Study	CT Chest	MRI	Echocardiogram	CT Chest	CT Chest/Abdomen/Pelvis
Date of Study	5 March 2021	17 March 2016	23 December 2013	7 January 2014	31 July 2019
Reason for Study	Chest Pain	Chest Pain	Fall	Chest Pain	Chest and Back Pain
Cardiovascular Comorbidities	HTN, AFib, Bradycardia, Dual chamber cardiac pace maker	HTN, AFib, HLD, CAD	HTN, Healed Des dissection	Asc AA	HTN, AFib, HLD, CAD, CHF
Myeloproliferative Neoplasms	Thrombocythemia, Anemia	Anemia	--	DVT	DVT/PE
Other Comorbidities	GERD	BPH, RUL nodule, BCC	--	CKD, Gigantism (GH-producing adenoma, s/p resection and RT), Pre-B ALL, Spontaneous colon perforation, May-Thurner syndrome, UTI, Hypoxia, Avascular necrosis hip, OSA, Orthostatic hypotension, SSS, BCC, Interferon therapy.	BPH, GERD, CKD, COPD
Surgical History	CABGx3, Asc AA s/p repair	Asc AA s/p AVR	Asc AA s/p repair	--	--

Legend: This table displays the clinical characteristics of the patients. Asc AA: ascending aortic aneurysm. AFib: atrial fibrillation. AVR: aortic valve replacement. BCC: basal cell carcinoma. BPH: benign prostate hyperplasia. CABG: coronary artery bypass grafting. CAD: coronary artery disease. CKD: chronic kidney disease. COPD: chronic obstructive pulmonary disease. Des: descending. DVT: deep venous thrombosis. GERD: gastroesophageal reflux disease. HLD: hyperlipidemia. HTN: hypertension. OSA: obstructive sleep apnea. PE: pulmonary embolism. RUL: right upper lobe. SSS: sick sinus syndrome. UTI: urinary tract infection.

**Table 2:** Characteristics of Patients with JAK2-V617F Somatic Variant without a Thoracic Aortic Aneurysm.

Characteristic	Patient 1	Patient 2	Patient 3	Patient 4	Patient 5	Patient 6	Patient 7
Sex	Male	Male	Female	Male	Male	Male	Male
Age	46	76	76	52	62	58	83
Weight (kg)	128.8	71.9	96.2	79.4	126	111.4	88.4
Height (m)	1.7	1.77	1.6	1.7	1.85	1.99	1.82
Aortic Size (cm)	2.9	3.2	3.5	--	3.6	--	2.9
JAK2 V617F Level in Blood	31.9%	39.8%	36%	30%	20%	11.8%	12%
Smoking History	Current	Never	Former	Never	Former	Never	Former
Diagnostic Imaging Study	Echo	Echo	Echo	--	Echo	--	Echo
Date of Study	9 April 2015	19 May 2014	16 February 2023	--	9 October 2023	--	9 April 2015
Cardiovascular Comorbidities	HTN, NICM, NSVT, CHF	--	Afib, HTN, PVD, HLD	HTN	AFib	HTN, HLD	HTN
MPN Comorbidities	Thrombocytosis	Myelofibrosis	MEN, Polycythemia Vera	Polycythemia vera	DVT/PE	Personal and family history of prostate cancer	Prostate cancer
Other Comorbidities	GERD, Diverticulitis	CKD, GERD, VWD	CKD, Colon cancer, OSA, Parathyroid tumor, CVA	Renal cell carcinoma, GERD, Pancreatitis, Budd–Chiari syndrome, Barrett’s esophagus	AKI, DM, OSA, TIA, Factor V Leiden	GERD	CVA

Legend: This table displays the clinical characteristics of the patients. AFib: atrial fibrillation. AKI: Acute kidney injury. CHF: Congestive heart failure. CKD: chronic kidney disease. CVA: Cerebrovascular disease. DM: Diabetes mellitus. DVT: deep venous thrombosis. GERD: gastroesophageal reflux disease. HLD: hyperlipidemia. HTN: hypertension. MPN: Myeloproliferative neoplasm. NICM: Non-ischemic cardiomyopathy. NSVT: Non-sustained ventricular tachycardia. OSA: obstructive sleep apnea. PE: pulmonary embolism. PVD: Peripheral vascular disease. TIA: Transient ischemic attack. VWD: von Willebrand Disease.

**Table 3:** Effect of JAK2 V617F on Circulatory System Phenotypes.

Category	Phenotype	*p*-Value	Effect Size (SE)	Number of Samples
Circulatory System	Nonrheumatic Aortic Valve Disorders	0.61	0.050 (0.098)	275/400,663
Circulatory System	Abdominal Aortic Aneurysm	0.63	−0.026 (0.054)	930/398,704
Circulatory System	Atherosclerosis of Aorta	0.73	−0.034 (0.096)	286/398,706
Circulatory System	Aortic Valve Disease	0.85	0.0086 (0.046)	1279/400,663
Circulatory System	Thoracic Aortic Aneurysm	0.92	0.0040 (0.043)	1457/398,705

Legend: This table displays the effect size of the *JAK2* V617F variant on various circulatory system phenotypes from the UKBB and FinnGen databases. SE: standard error.

## Data Availability

The data underlying this article will be available upon reasonable request to the corresponding author.
